# Standardisation and evaluation of a quantitative multiplex real-time PCR assay for the rapid identification of *Streptococcus pneumoniae*

**DOI:** 10.15172/pneu.2015.6/559

**Published:** 2015-12-01

**Authors:** Feroze A. Ganaie, Vandana Govindan, K. L. Ravi Kumar

**Affiliations:** Department of Microbiology, Kempegowda Institute of Medical Sciences, Hospital and Research Centre, K.R Road, V.V Puram, Bangalore, 560004 India

**Keywords:** multiplex real-time PCR, *Streptococcus pneumoniae*, pneumolysin, autolysin, pneumococcal surface adhesin A

## Abstract

Rapid diagnosis of *Streptococcus pneumoniae* can play a significant role in decreasing morbidity and mortality of infection. The accurate diagnosis of pneumococcal disease is hampered by the difficulties in growing the isolates from clinical specimens and also by misidentification. Molecular methods have gained popularity as they offer improvement in the detection of causative pathogens with speed and ease. The present study aims at validating and standardising the use of 4 oligonucleotide primer-probe sets (pneumolysin [*ply*], autolysin [*lytA*], pneumococcal surface adhesion A [*psaA*] and Spn9802 [DNA fragment]) in a single-reaction mixture for the detection and discrimination of *S. pneumoniae*. Here, we validate a quantitative multiplex real-time PCR (qmPCR) assay with a panel consisting of 43 *S. pneumoniae* and 29 non-pneumococcal isolates, 20 culture positive, 26 culture negative and 30 spiked serum samples. A standard curve was obtained using *S. pneumoniae* ATCC 49619 strain and glyceraldehyde 3-phosphate dehydrogenase (*GAPDH*) gene was used as an endogenous internal control. The experiment showed high sensitivity with lower limit of detection equivalent to 4 genome copies/µl. The efficiency of the reaction was 100% for *ply*, *lytA*, Spn9802 and 97% for *psaA*. The test showed sensitivity and specificity of 100% with culture isolates and serum specimens. This study demonstrates that qmPCR analysis of sera using 4 oligonucleotide primers appears to be an appropriate method for the genotypic identification of *S. pneumoniae* infection.

## 1. Introduction

*Streptococcus pneumoniae* is an important bacterial pathogen in humans that is recognised as a major cause of pneumonia, meningitis, sinusitis, otitis media, and as an uncommon cause of a variety of other infectious diseases [1]. Pneumococcal disease is under-reported, as only a small portion of presumptive cases can be confirmed by conventional techniques. Isolation of *S. pneumoniae* from blood occurs in only 20–30% of adult cases of pneumococcal pneumonia and less than 10% of cases among children [2,3]. Even when present in blood, *S. pneumoniae* may be missed due to a low density of the pathogen, the fastidious nature of the organism, and previous administration of antibiotics [4]. Serologic assays for both antibody and antigen detection lack specificity and sensitivity [5,6].

Accurate and rapid assays are indispensable for prompt diagnosis and effective therapy. Molecular assays with increased sensitivity and specificity are inherently beneficial for detection of infectious agents and are not abated by non-viable organisms [7]. Various molecular assays, including loop-mediated isothermal amplification method (LAMP) [8], DNA probe test [9], and TaqMan® quantitative real-time polymerase chain reaction (qPCR) assay [10] have been developed and employed to assist investigations.

Studies have revealed that genes encoding pneumolysin (*ply*) [10], autolysin (*lytA*) [7], pneumococcal surface adhesion A (*psaA*) [11], penicillin binding protein [12], and manganese-dependent superoxide dismutase [13] have been targeted for identification of *S. pneumoniae* by PCR-based assays. The *ply* and *lytA* genes are among the more common gene targets used for screening *S. pneumoniae*. Although *lytA* is present in *S. pneumoniae* as well as related species such as oral streptococci, the *lytA* gene has sufficient variability that properly designed primers afford specificity for *S. pneumoniae* [14,15].

Amongst pneumococcus-like viridans group streptococci, a newly recognised species classified as *S. pseudopneumoniae* which is positive by AccuProbe assay has been described and characterised [2]. Studies have shown that *lytA* and *psaA* gene sequences can reliably distinguish *S. pneumoniae* from *S. pseudopneumoniae* [16]. Further, Suzuki et al. [17] developed a highly specific Spn9802 primer set for *S. pneumoniae* in order to discriminate *S. pneumoniae* from pneumococcus-like oral streptococci harbouring the *ply* and *lytA* genes; however, these Spn9802 primers may not discriminate *S. pseudopneumoniae* [15]. For the identification of pneumococci, PCR tests have been applied by various researchers, however, there are reports of ambiguous results [5]. Such ambiguities are perhaps not surprising given the diversity that exists within the organisms that are closely related to pneumococci. It is evident that these organisms can harbour pneumococcal virulence determinants such as *ply* and *lytA* genes that are notionally used for diagnosis for pneumococci [18]. So, straightforward amplification of *ply* by PCR or in combination with *lytA* is unable to resolve the identification of strains with equivocal reactions for serotype, optochin, or bile solubility [5,18]. The development of a diagnostic test based upon single-target identification is an ambitious hope in a clinical laboratory setting. Given the genetic plasticity of the pneumococcus and its naturally transformable relatives, the combination of 3 or more unique loci is needed to reduce the possibility of false positives [5].

Accurate detection of pneumococcal infection is of importance to estimate disease burden, tracking changes in the epidemiology of the disease and to assess the effectiveness of currently used vaccines. In the present work, we have standardised and evaluated the quantitative multiplex real-time PCR (qmPCR) system for the accurate and rapid identification of *S. pneumoniae*.

## 2. Methods

### 2.1 Bacterial isolates

*S. pneumoniae* ATCC 49619 (American Type Culture Collection, USA) was used as a reference strain for optimisation of the assay. Twenty-three characterised and typed *S. pneumoniae* strains were procured from Statens Serum Institute, Denmark (serotypes 1, 2, 3, 4, 5, 6A, 6B, 7F, 8, 9N, 9V, 10A, 11A, 12F, 14, 15B, 17F, 18C, 19F, 19A, 20, 22F, 23F). Twenty invasive *S. pneumoniae* strains isolated from whole blood samples of children with pneumonia and 29 negative control organisms were obtained from Central Research Laboratory, Kempegowda Institute of Medical Sciences, Hospital and Research Centre, Bangalore. These organisms were cultured under conditions appropriate for each species [19].

The isolates were identified as *S. pneumoniae* by colony morphology, alpha haemolysis, Gram stain, optochin sensitivity, and bile solubility tests by standard methods [20]. The identification was confirmed by automated MicroScan WalkAway-40 (Siemens Healthcare Diagnostics Ltd, UK) system with rapid identification panels [21].

### 2.2 Serum specimens

Twenty serum samples from patients who were positive for pneumococcal blood culture, 26 from patients who were negative for pneumococcal blood culture, and 30 from healthy subjects were included in the study. The DNA extracted from the serum samples of healthy subjects was subsequently spiked with 4.29 × 10^2^ copies/µl or 1 pg/µl of *S. pneumoniae* ATCC 49619 genomic DNA. Serum samples were sourced from the Pneumococcal Research Division, Central Research Laboratory, Kempegowda Institute of Medical Sciences, Hospital and Research Centre, Bangalore.

### 2.3 DNA extraction/or qmPCR analysis

DNA extraction from isolates and serum specimens was performed using QIAamp DNA Mini Kit with automated DNA extracter, QIAcube (Qiagen, Germany), as per manufacturer’s protocol. Briefly, a loopful of the overnight growth from a blood agar plate was suspended in 180 µl of suspension buffer followed by 20 µl proteinase K and incubated at 56°C for 30 min. For serum, 200 µl of lysis buffer was added to 200 µl of clinical material followed by 20 µl of proteinase K and incubated at 70°C for 10 min. After washing steps, DNA was eluted in 100 µl of elution buffer and stored at −20°C.

#### 2.3.1 Quantification and quality determination of extracted DNA

Quantification and quality of the extracted DNA was determined spectrophotometrically at absorbance 260 nm using Nanodrop 2000 (Thermo Fisher Scientific, USA). The optical density of extracted DNA was measured at the wavelengths of 260 and 280 nm. The DNA purity was estimated by measuring the ratio between the absorbance values.

### 2.4 Primer and hydrolysis probe oligonucleotide design

The oligonucleotide sequences for *ply, lytA, psaA*, Spn9802, and glyceraldehyde 3-phosphate dehydrogenase (*GAPDH*) primer-probe sets were obtained from previously published data [2,22,23] and the sequences available in the GenBank database (Table 1). For the Spn9802 probe sequence, extra ATC and TAC bases were added at the 5′ and 3′ ends, respectively. All sequences were analysed for specificity and PCR suitability using the National Centre for Biotechnology Information (NCBI) Primer-BLAST software (https://doi.org/blast.ncbi.nlm.nih.gov/Blast.cgi). Hydrolysis-probes were labeled with the appropriate 5′ reporter dyes (FAM, CAL Fluor Red 610, CAL Fluor Orange 560, Quasar 705, and Quasar 670) and 3′ Black Hole Quencher dyes (BHQ-1, BHQ-2, BHQ-1-plus). BHQ-1-plus quencher was utilised on the Spn9802 probe to improve duplex stability and enhance target specificity. Primers and hydrolysis probes were synthesised from Biosearch Technologies, USA.

**Table 1:** Primer and probe sequences for quantitative multiplex real-time PCR

Oligonucleotide primer	Sequence	Product size (bp)	GenBank accession no.
*ply*-forward	5′-GCTTATGGGCGCCAAGTCTA-3′	78	NC_003028.3
*ply*-reverse	5′-CAAAGCTTCAAAAGCAGCCTCTA-3′		
*ply*-probe	5′-Quasar 705-CTCAAGTTGGAAACCACGAGTAAGAGTGATGAA-3′-BHQ-2		
*lytA*-forward	5′-ACGCAATCTAGCAGATGAAGCA-3′	75	NC_003028.3
*lytA*-reverse	5′-TCGTGCGTTTTAATTCCAGCT-3′		
*lytA*-probe	5′-FAM-GCCGAAAACGCTTGATACAGGGAG-3′-BHQ-1		
*psoA*-forward	5′-GCCCTAATAAATTGGAGGATCTAATGA-3′	114	U53509.1
*psoA*-reverse	5′-GACCAGAAGTTGTATCTTTTTTTCCG-3′		
*psoA*-probe^a^	5′-CAL Fluor Red 610-CTAGCACATGCTACAAGAATGATTGCAGAAAGAAA-3′-BHQ-2		
Spn9802-forward	5′-AGTCGTTCCAAGGTAACAAGTCTAG-3′	157	AE005672.3
Spn9802-reverse	5′-ACCAACTCGACCACCTCTTTC-3′		
Spn9802-probe^b^	5′-CAL Fluor Orange 560-ATCAGATTGAAGCTGATAAAACGATAC-3′-BHQ-1 *plus*		
*GAPDH*-forward	5′-GAAGGTGAAGGTCGGAGT-3′	226	BC083511.1
*GAPDH*-reverse	5′-GAAGATGGTGATGGGATTTC-3′		
*GAPDH*-probe	5′-Quasar 670-CTCAAGTTGGAAACCACGAGTAAGAGTGATGAA-3′-BHQ-2		

ply, pneumolysin gene; *lytA*, autolysin gene; *psaA*, pneumococcal surface adhesin A gene; *GAPDH*, glyceraldehyde 3-phosphate dehydrogenase gene; Spn9802, *Streptococcus pneumoniae* DNA fragment

^a^*psaA* probe is designed to bind to the reverse strand of the amplicon

^b^Spn9802 probe sequence modified to BHQ-1-*plus* probe and extra ATC and TAC bases were added at the 5′ and 3′ ends, respectively.

The Rotor-Gene Q (Qiagen) thermocycler has 5 preset channels: Green, Yellow, Orange, Red and Crimson, with the detection range from 450 to 712 nm. Each channel detects reporter dyes that emit light at a particular wavelength. Based on the quenching range of BHQ-1 (480–580 nm), BHQ-2 (559–670 nm), emission wavelength of the reporter dye and detection optics of the instrument, we confirmed that each selected reporter dye is compatible and detected by respective detection channels.

### 2.5 qmPCR assay

A 20X primer-probe mix comprising 10 µM of each forward primer, 10 µM of each reverse primer, and 4 µM of each hydrolysis probe was prepared in DNase/RNase free water (Qiagen) from 100 µM stock solutions of each primer and hydrolysis probe. PCR was performed in 25 µl volumes using the 2X Rotor-Gene Multiplex PCR Master Mix (Qiagen) containing HotStar TaqPlus DNA polymerase (Qiagen), MgCl_2_ and deoxynucleotide triphosphates (dNTPs: dATP, dCTP, dGTP, dTTP), 20X primer-probe mix, template DNA and water, as described in Table 2. Final optimal forward/reverse primer and probe concentrations were 0.5 µM and 0.2 µM, respectively. The qmPCR assays were performed on the Rotor-Gene® Q (Qiagen) thermocycler using 200 µl PCR tubes and 36 well rotor. Optimal performance of the assay was achieved by following the thermocycling conditions as recommended by the manufacturer. It consisted of 1 cycle of denaturation at 95°C for 5 min, followed by 45 cycles of denaturation at 95°C for 30 s and combined annealing/extension step at 60°C for 15 s. Data analysis was performed by using Rotor-Gene® Q software (Qiagen). Analysis settings (threshold values, background settings, dynamic tube normalisation, outlier removal) for every reporter dye detection channel were adjusted in every run to obtain accurate quantification data as suggested by the manufacturer and as has been described elsewhere [24].

**Table 2:** Quantitative multiplex real-time PCR (qmPCR) set-up

Component	Volume/reaction	Final concentration
2X Rotor-Gene Multiplex PCR Master Mix	12.5 µl	1X
20X primer-probe mix *5^a^	1.25 µl*5	0.5 µM forward primer*5
		0.5 µM reverse primer *5
		0.2 µM probe *5
Template DNA	5 µl	<100 ng/reaction
DNase/RNase free water	1.25 µl	
Total reaction volume	25 µl	

^a^20X primer-probe mix for all 5 primer-probe sets was prepared and added individually in a single-reaction

#### 2.5.1 Standard curve and lower limit of detection

**Figure 1:**
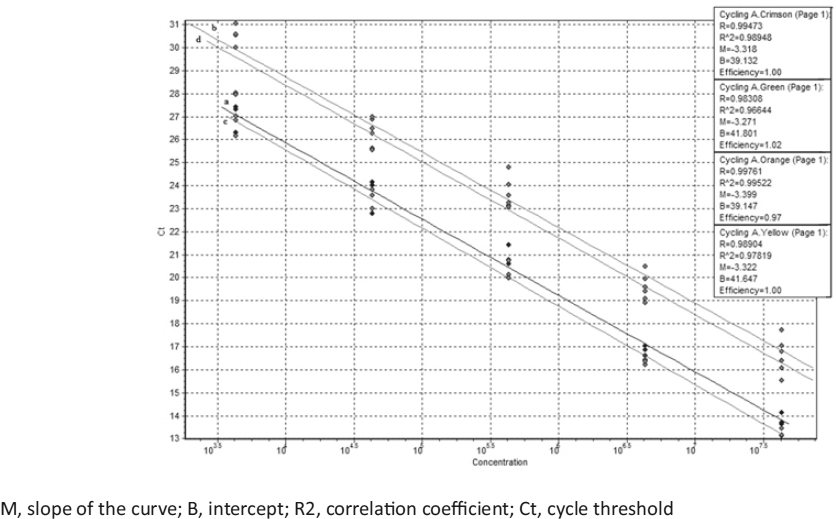
Graph showing overlay of standard curves generated by different detection channels: a = Cycling A Crimson (*ply*), b = Cycling A Green (*lytA*), c = Cycling A Orange (*psaA*), d = Cycling A Yellow (Spn9802)

Standard curves (Figure 1) for each gene target in the qmPCR assay was generated by 10-fold serial dilutions of *S. pneumoniae* ATCC 49619 DNA equivalent to 4.29 × 10^5^ to 4.29 × 10^1^ genome copies/µl (1 ng/µl to 100 fg/µl). Each standard dilution was run in triplicate.

For assessments of the lower limit of detection (LLD), serial 10-fold dilutions equivalent to from 4.29 × 10^5^ to 0.4 genome copies/µl (1 ng/µl to 1 fg/µl) of purified DNA were prepared and the aliquots were tested by the *ply-lytA*-, *psaA*-, and Spn9802-specific primer-probe sets in multiplex reaction [2,7].

#### 2.5.2 Sensitivity and specificity on culture isolates

Sensitivity of the qmPCR assay was determined by testing 10 ng/µl of DNA from 43 confirmed *S. pneumoniae* isolates. Specificity determination was made by testing 10 ng/µl of DNA extracted from 29 non-pneumococcal isolates in the multiplex reaction. We could not test *S. pseudopneumoniae* due to non-availability of the strain.

#### 2.5.3 Sensitivity and specificity on serum specimens

DNA extracted from 30 serum samples of healthy subjects spiked with 1 pg/µl of *S. pneumoniae* ATCC 49619 genomic DNA corresponding to 4.29 × 10^2^ genome copies/µl and 20 *S. pneumoniae* culture positive serum samples were tested by the qmPCR for sensitivity determination. Twenty-six culture-negative serum samples were examined by the qmPCR for specificity determination. The extracted DNA from each sample (5 µl) was used as a template. *GAPDH*-specific primer-probe set was used to amplify the endogenous internal control in multiplex reactions.

A no template control (NTC) was included in every run. Positive samples were defined as those which showed amplification for ≥3 target specific sequences and LLD of ≥4 genome copies/µl. Culture and serum specimens were run in duplicates.

### 2.6 Ethics statement

The study was approved by the Kempegowda Institute of Medical Science independent ethics committee (Approval ID: ECR/216/Inst/Kar/2013). The study was conducted according to the guidelines and recommendations of Good Clinical Practice and the Declaration of Helsinki. Written informed consent was obtained from each participant or legal guardian as applicable.

### 2.7 Statistical analysis

Sensitivity and specificity of qmPCR on culture and serum specimens were calculated using MedCalc Software bvba version 15.6 (Belgium).

## 3. Results

### 3.1 qmPCR standard curves

**Figure 2:**
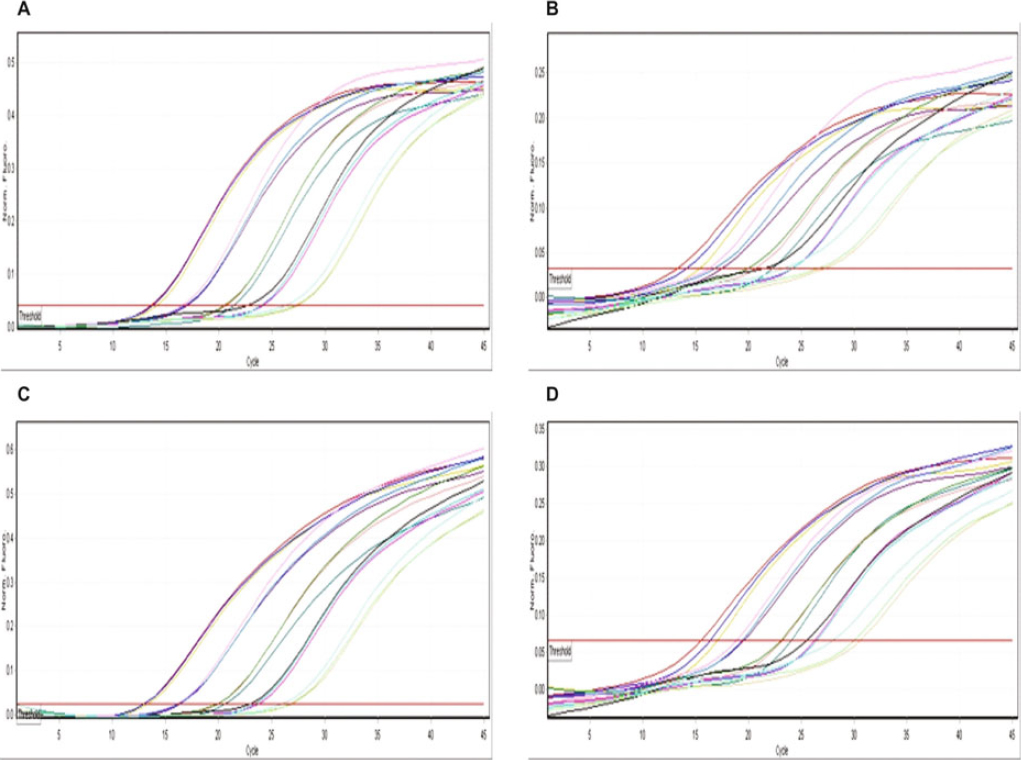
Quantitative multiplex real-time PCR for *Streptococcus pneumoniae* (ATCC 49619) showing amplification profile for Cycling A: A) Crimson (*ply*), B) Green (*lytA*), C) Orange (*psaA*) and D) Yellow (Spn9802). The X and Y axes represent amplification cycles and fluorescence units, respectively

A linear standard curve was acquired for each of the *ply, lytA, psaA* and Spn9802 primer-probe sets within the qmPCR between 4.29 × 10^5^−4.29 × 10^1^ genome copies/µl (1 ng/µl–100 fg/µl) of DNA from the reference strain *S. pneumoniae* ATCC 49619. The slope of the curves ranged from −3.399 to −3.271 with the R^2^ value ≥0.97. The efficiency of the qmPCR assay for all the detection channels were very similar and ranged from 97% to 100% (0.97–1.0). The former was contributed by *psaA*, and the latter by *ply*, *lytA* and Spn9802. The amplification profile for each target is provided in Figure 2.

The qmPCR assay illustrated LLD equivalent to 4 genome copies/µl or 20 genome copies/reaction for *ply-, lytA-, psaA-* and Spn9802-specific primer-probe sets. There was an increase in quantification cycle (Cq) values with the decrease in template concentration in subsequent reactions.

### 3.2 Standardisation of qmPCR on culture isolates

The qmPCR was positive for all 43 *S. pneumoniae* strains, representing 23 different serotypes, indicating 100% sensitivity for known isolates. The specificity of each of the specific primer-probe sets was evaluated and the specificities were compared by amplifying DNA extracted from 29 strains of non-pneumococcal bacteria. These strains represented several genera of Gram-positive and Gram-negative bacteria, some of which inhabit the oral cavity (Table 3). There was no amplification of any non-pneumococcal bacteria by any of the primer-probe sets (*ply, lytA, psaA*, and Spn9802) indicating 100% specificity for this cross-reactivity panel (Table 4).

**Table 3:** Cross-reactivity panel: negative control organisms

Genus	Species or serovar(s)(subtypes)
*Homo*	*H. sapiens*
*Streptococcus*	*S. agalactiae, S. mitis, S. equi, S. pyogenes, S. sanguis, S. epidermidis*
*Shigella*	*S. flexneri, S. sonii, S. boydii, S. dysenteriae*
*Salmonella*	*S*. Typhi-I, *S. paratyphi, S. typhimurium*
*Vibrio*	*V. Inaba, V. halioticoli, V. ordalii*
*Enterococcus*	*E. fecalis, E. faecium, E. solitarius*
*Staphylococcus*	*S. aureus*, Coagulase negative *S. aureus*
*Proteus*	*P. vulgaris*
*Corynebacteria*	*C. diphtheria*
*Escherichia*	*E. coli H1*
*Acinetobacter*	*A. baumanni*
*Klebsiella*	*K. pneumoniae*
*Citrobacter*	*C. freundii*
*Pseudomonas*	*P. aeuroginosa*

**Table 4:** Sensitivity and specificity of quantitative multiplex real-time PCR (qmPCR)

Specimen	No. of samples	Culture positive for pneumococci (%)	Culture negative for pneumococci (%)	qmPCR positive^a^ (%)	qmPCR negative^b^ (%)
*S. pneumoniae-culture* positive isolates	43	43 (100)	0	43 (100)	0
Non-pneumococcal isolates	29	0	29 (100)	0	29 (100)
Spiked serum samples	30			30 (100)	0
Culture-positive serum samples	20	20 (100)	0	20 (100)	0
Culture-negative serum samples	26	0	26 (100)	0	26 (100)

^a^Sensitivity of qmPCR in pneumococcal culture-positive isolates and serum specimens was 100%

^b^Specificity of qmPCR in pneumococcal culture-negative isolates and serum specimens was 100%

### 3.3 Standardisation of qmPCR on serum specimens

All 30 spiked DNA extracts were qmPCR positive at the expected concentration (4.29 × 10^2^ genome copies/µl or 1 pg/µl) demonstrating that the DNA extraction eluted from serum samples was free from PCR inhibitors.

All 20 serum samples previously shown as blood culture positive for *S*. *pneumoniae* were positive for each target (*ply, lytA, psaA*, and Spn9802) in the qmPCR, indicating 100% sensitivity. Quantification data of the 20 *S. pneumoniae* positive serum samples for each target is described in Table 5. All 26 serum samples, previously shown as blood culture negative for *S. pneumoniae*, were negative for each target (*ply, lytA, psaA*, and Spn9802) in the qmPCR, indicating 100% specificity (Table 4). Endogenous internal control *GAPDH* was amplified in all the multiplex reactions along with the *S. pneumoniae*-specific primer-probe sets.

**Table 5:** Quantification data of the 20 *Streptococcus pneumoniae*-positive serum samples

Sample ID	Serotype	Ct-*ply*	Calc Conc (genome copies/µl)	Inference	Ct-*lytA*	Calc Conc (genome copies/µl)	Inference	Ct-*psaA*	Calc Cone (genome copies/µl)s	Inference	Ct-Spn9802	Calc Cone (genome copies/µl)s	Inference	Ct-*GAPDH*	Calc Conc (genome copies/µl)	Inference
Serum-1	Unknown	28.29	1260	Positive	31.57	1005	Positive	28.83	1113	Positive	30.06	770	Positive	38.67	98	Positive
Serum-2	Unknown	29.75	660	Positive	31.8	323	Positive	30.07	412	Positive	30.57	213	Positive	33.01	312	Positive
Serum-3	Unknown	27.95	1890	Positive	29.9	1276	Positive	28.2	1430	Positive	28.51	995	Positive	35.17	278	Positive
Serum-4	Unknown	28.36	745	Positive	29.89	812	Positive	28.9	792	Positive	28.54	211	Positive	35.65	301	Positive
Serum-5	Unknown	30.42	475	Positive	32.02	320	Positive	31.28	305	Positive	31.2	66	Positive	30.09	411	Positive
Serum-6	Unknown	30.11	412	Positive	30.87	297	Positive	30.99	201	Positive	30.39	103	Positive	33.16	231	Positive
Serum-7	Unknown	30.57	616	Positive	33.33	677	Positive	31.52	811	Positive	31.68	313	Positive	34.86	197	Positive
Serum-8	Unknown	30.25	928	Positive	32.11	829	Positive	31	896	Positive	31.03	308	Positive	38.79	57	Positive
Serum-9	Unknown	27.1	1101	Positive	29.9	980	Positive	28.04	935	Positive	28.15	543	Positive	33.39	111	Positive
Serum-10	Unknown	27.48	876	Positive	30.7	1122	Positive	28.07	996	Positive	29.31	401	Positive	35.57	78	Positive
Serum-11	Unknown	28.74	1323	Positive	31.7	1044	Positive	29.58	1121	Positive	30.86	347	Positive	39	34	Positive
Serum-12	Unknown	29.78	801	Positive	32.04	598	Positive	30.29	721	Positive	30.89	421	Positive	37.35	59	Positive
Serum-13	Unknown	30.48	668	Positive	33.21	422	Positive	30.86	537	Positive	31.91	213	Positive	40.19	12	Positive
Serum-14	Unknown	30.1	373	Positive	33.33	394	Positive	30.27	456	Positive	31.49	218	Positive	39.52	23	Positive
Serum-15	Unknown	27.68	511	Positive	29.95	606	Positive	28.02	727	Positive	29.07	398	Positive	38.53	33	Positive
Serum-16	Unknown	30.59	123	Positive	32.84	66	Positive	31.25	96	Positive	31.51	54	Positive	37.44	102	Positive
Serum-17	Unknown	30.54	554	Positive	33.95	412	Positive	31.45	543	Positive	32.78	194	Positive	40.27	45	Positive
Serum-18	Unknown	29.02	728	Positive	30.95	631	Positive	29.45	812	Positive	29.72	509	Positive	35.1	219	Positive
Serum-19	Unknown	30.05	303	Positive	32.68	78	Positive	30.14	294	Positive	31.23	166	Positive	38.71	133	Positive
Serum-20	Unknown	27.92	280	Positive	29.62	142	Positive	28.81	194	Positive	29.26	102	Positive	38.82	68	Positive

Ct, cycle threshold; Calc Conc, calculated concentration

## 4. Discussion

Real-time PCR assays have immense potential to serve as sensitive diagnostic tests for the detection of invasive *S*. *pneumoniae*. Currently, there is no simple and dependable method to assess its performance; hence, the diagnostic capability of qPCR should be critically evaluated to acquire reliable results [15,25]. In this study, we evaluated a 4 target qmPCR for accurate detection of *S. pneumoniae* in culture and serum specimens.

Our qmPCR targeting *ply, lytA, psaA*, and Spn9802 sequences had 100% sensitivity for detecting *S. pneumoniae* in culture isolates. The assay also had 100% specificity against a cross-reaction panel of 29 organisms representing diverse genera, including 6 streptococcal species. The present study shows greater sensitivity and specificity than reported by Falquera et al. [26] (78% sensitivity, 93% specificity), Toikka et al. [27] (44% sensitivity, 100% specificity), and Michelow et al. [28] (92% sensitivity, 95% specificity). However, our results are in concordance with the findings of Carvalho et al. [2], McAvin et al. [7], and Messmer et al. [16], who also reported 100% sensitivity and specificity.

qPCR for pneumococci in serum has been reported to be challenging due to the presence of inhibitors in blood and the low number of genomic copies [4,29]. In the present study, qmPCR testing of 30 spiked DNA extracts and 20 pneumococcal culture-positive serum specimens correlated completely with the culture results, indicating 100% sensitivity. High correlation results were also reported by Bayram et al. [30] (97.2%) and Stralin et al. [23] (94%). None of the 26 culture-negative serum samples were positive by qmPCR, suggesting 100% specificity. Similar results were reported by Ismail et al. [14], Carvalho et al. [2] and McAvin et al. [7].

In the present study, all the primer-probe sets showed a lower limit of detection equivalent to 4 genome copies/µl. This is similar to that reported by McAvin et al. [7] and Rudolph et al. [31]. Scott et al. [32] and Carvalho et al. [2] reported 1 genome copy/µl and <10 genome copies/µl, respectively. Even though the PCR could detect as little as one genome copy of target DNA as has been described by Scott et al. [32], the sensitivity of pneumococcal PCR may be poor in clinical evaluations due to the presence of inhibitors. *Taq* polymerase is highly sensitive to porphyrin inhibitors that are generated from the breakdown of haemoglobin. Most of the inhibitors can be eliminated by an efficient DNA extraction method. In our study, human *GAPDH* used as an endogenous internal control amplified in all the reactions, suggesting optimal DNA extraction, qmPCR efficiency, and absence of inhibitors.

The efficiency, accuracy, sensitivity, and dynamic range of the PCR assay is determined by the standard curve which is independent of variables associated with the sample preparation. The standard curves generated in this study showed efficiency of 97% to 100%. Similar findings were reported by Carvalho et al. [2]. The high efficiency of this protocol signifies that the amount of PCR product is doubling during each cycle and there is an absence of PCR inhibitors.

By targeting the *ply* gene, *S. pneumoniae* DNA in blood samples has been detected with sensitivity ranging from 35% to 100% [15,33]. A matter of concern for the *ply* PCR is that it is unable to distinguish *S. pneumoniae* from other streptococcal species [15]. In view of the low specificity of the *ply* PCR, the *lytA* PCR has been used and found to have higher specificity [2,34], and pneumococcal DNA was not detected in the blood of healthy subjects irrespective of carrier status [35]. Sequencing and high resolution DNA typing of *S. pneumoniae* illustrated conservation of the *lytA* gene. It has been shown that *lytA* differentiates *S. pneumoniae* from genotypically related species [12]. Monoclonal antibody studies suggest that *psaA* is expressed in all 90 serotypes of *S. pneumoniae*, and PCR-restriction fragment length polymorphism analysis of the 23 vaccine serotypes demonstrated the conservation of the gene [11]. Recently a new *S. pneumoniae* specific target, the gene fragment Spn9802, has been reported to discriminate *S. pneumoniae* from pneumococcus-like strains [17].

Application of multiplex real-time PCR in serum specimens has emerged as a valuable clinical diagnostic tool that offers an opportunity to readdress the problem of the diagnosis of *S. pneumoniae* infections. The problems associated with microorganisms that are low in number, difficult to culture, or antigenically similar has been minimised with the advancement of sensitive and specific multiplex PCR assays [30,36]. It has several positive outcomes over the singleplex assay as it reduces test costs, eliminates well-to-well variability, conserves precious samples, increases test throughput, and improves turnaround times [37].

Multiplex PCR assays are popularly adopted for simultaneous detection of various pathogens in the clinical specimens. In the present protocol, multiple specific primer-probe sets were used for detection of a single pathogen to eliminate the spurious negative and positive results. Our study establishes that—with high sensitivity, specificity, and rapidity—the qmPCR assay is a valid platform for detection of pneumococci from serum specimens in clinical laboratories. Finally, the present methodology has broader applications beyond the scope of the present study that demands further investigation. Future studies with large sample sizes should seek to replicate our findings across different geographical locations, particularly in regions with high prevalence of pneumococcal infections.

The qmPCR assay targeting *ply*, *lytA*, and *psaA* genes and Spn9802 DNA fragment is a sensitive and specific assay for the rapid identification of *S. pneumoniae*. This technology should offer an added advantage when it is used in conjunction with other assays for pneumococcal disease diagnosis. The efficacy of the DNA extraction procedure, degree of sensitivity, and specificity supports the use of this procedure for the direct detection of *S. pneumoniae* in patient specimens.
