# Erratum to: Standardisation and evaluation of a quantitative multiplex real-time PCR assay for the rapid identification of Streptococcus pneumonia

**DOI:** 10.1186/s41479-017-0034-1

**Published:** 2017-07-06

**Authors:** Feroze A. Ganaie, Vandana Govindan, K. L. Ravi Kumar

**Affiliations:** Department of Microbiology, Kempegowda Institute of Medical Science, Hospital and Research Centre, Bangalore, -560004 India

## Erratum

In the publication of this article [1], there was an error in Table 1 which has a wrong sequence value at the GAPDH-probe.

The error:

‘5′-Quasar 670–CTCAAGTTGGAAACCACGAGTAAGAGTGATGAA-3′-BHQ-2’.

Should instead read:

‘5′-Quasar 670– CAAGCTTCCCGTTCTCAGCC-3′-BHQ-2 ‘.

This has now been included in this erratum.
